# The complete mitochondrial genome of *Pseudopimelodus schultzi* Dahl 1955 (Siluriformes, Pseudopimelodidae) and its phylogenetic position within Pseudopimelodidae

**DOI:** 10.1080/23802359.2021.1945974

**Published:** 2021-08-01

**Authors:** Hanwei Yang, Xiuhui Ma

**Affiliations:** School of Animal Science, Guizhou University, Guiyang, China

**Keywords:** Mitochondrial genome, *Pseudopimelodus schultzi*, next-generation sequencing

## Abstract

The complete mitochondrial DNA genome of *Pseudopimelodus schultzi* was first was determined in this study. The entire length of mitochondrial genome consists of 13 protein-codinggenes (PCG), 2 ribosomal RNA genes (rRNA), and 22 transfer RNA (tRNA) genes and control region. The nucleotide composition was made up of 32.2% A, 25.9% T, 26.7% C, and 15.2% G, respectively, indicating an A + T (58.1%)-rich feature. With the exception of 8 tRNA genes and NADH6, mitochondrial genes are encoded on the heavy strand, which was similar to that in other vertebrates. The results showed that the species of Pseudopimelodidae were gathered in the same branch. The phylogenies indicate monophyly of the genus *Batrochoglanis*, *Batrochoglanis* and *Microglanis*, respectively.

The family Pseudopimelodidae, commonly known as Bumblebee Catfish, belongs to Siluriformes, which is widely distributed in South America. There are 7 genera and 66 valid species in this family in fishbase, but only one mt DNA sequences of *Lophiosiluru salexandri* are available (Sato et al. 2018).

The phylogenetic position of Pseudopimelodidae remains controversial. Lundberg et al. (1991) demonstrated the monophyly of Pseudopimelodidae and considered its sistergroup relationship with Rhamdiinae (= Heptapteridae) to be weakly supported based on lip structure. Britto (2003) recovered it as related to Heptapteridae, whereas Diogo et al. (2004) considered it sister to a clade comprising Pimelodidae and Heptapteridae. Specimens of *Pseudopimelodus schultzi* Dahl 1955, distributed in South America: Magdalena River basin, Colombia,were collected from Huadiwan Flowers & Birds market in Guangzhou, China (N: 23°05′13.76″; E: 113°13′34.52″). Voucher specimens were preserved in 100% ethanol and deposited at School of Animal Science, Guizhou University (Fang Ren, 1569400241@qq.com) under the voucher number gzu20190025. The complete mitochondrial genomes were amplified from the genomic DNA using four overlapping amplification primers by long PCR methods. Then, complete mitochondrial DNA library was constructed using the “With- Bead” Method (Fisher et al. 2011) and sequenced on the Miseq sequencer (Illumina, Inc., San Diego, CA, USA).Contigs were assembled de novo with Trinity (Grabherr et al. 2011).In the present study, the complete mitochondrial DNA genome of *P. schultzi* was first reported. The complete mitochondrial sequence of *P. schultzi* had been deposited in the GenBank with an accession number MW959699.The entire length of mitochondrial genome is 16,455 bp and the nucleotide composition was made up of 32.2% A, 25.9% T, 26.7% C, and 15.2% G, respectively, indicating an A + T (58.1%)-rich feature. The mitochondrial sequence was annotated with the web-site based tool DOGMA (Wyman et al. 2004) and 22 tRNA genes were scanned by tRNAscan-SE (Lowe and Chan 2016). The mitogenome includes 22 transfer RNA genes, 2 ribosomal RNA genes, 13 protein-coding genes and one non-coding control region (D-loop). Total 86 bp intergenic nucleotides ranging from 1 to 30 bp were identified in the whole genome. Except for overlapped between NADH5 and NADH6 encoded on opposite strands, some overlaps were also appeared on 13 protein-coding genes encoded on the same strand.

The phylogenetic relationship of *P. schultzi* and other Pseudopimelodidae species was inferred using the neighbor-joining and maximum likelihood method in MEGA 7.0 (Kumar et al. 2016). The results showed that the species of Pseudopimelodidae were gathered in the same branch. The phylogenies indicate monophyly of the genus *Pseudopimelodus* with very high support values (BP = 94, Figure 1). The *Pseudopimelodus bufonius* was placed with *Pseudopimelodus charus* to form a sister group to *P. schultzi* in the phylogenetic tree. The phylogenies indicate monophyly of the genus *Batrochoglanis*, *Batrochoglanis* and *Microglanis*, respectively. This report could enrich the mitogenome resource of *P. schultzi* and it also seems to be useful for evolutionary and conservation studies on Pseudopimelodidae and Siluriformes fish species.

**Figure 1.**
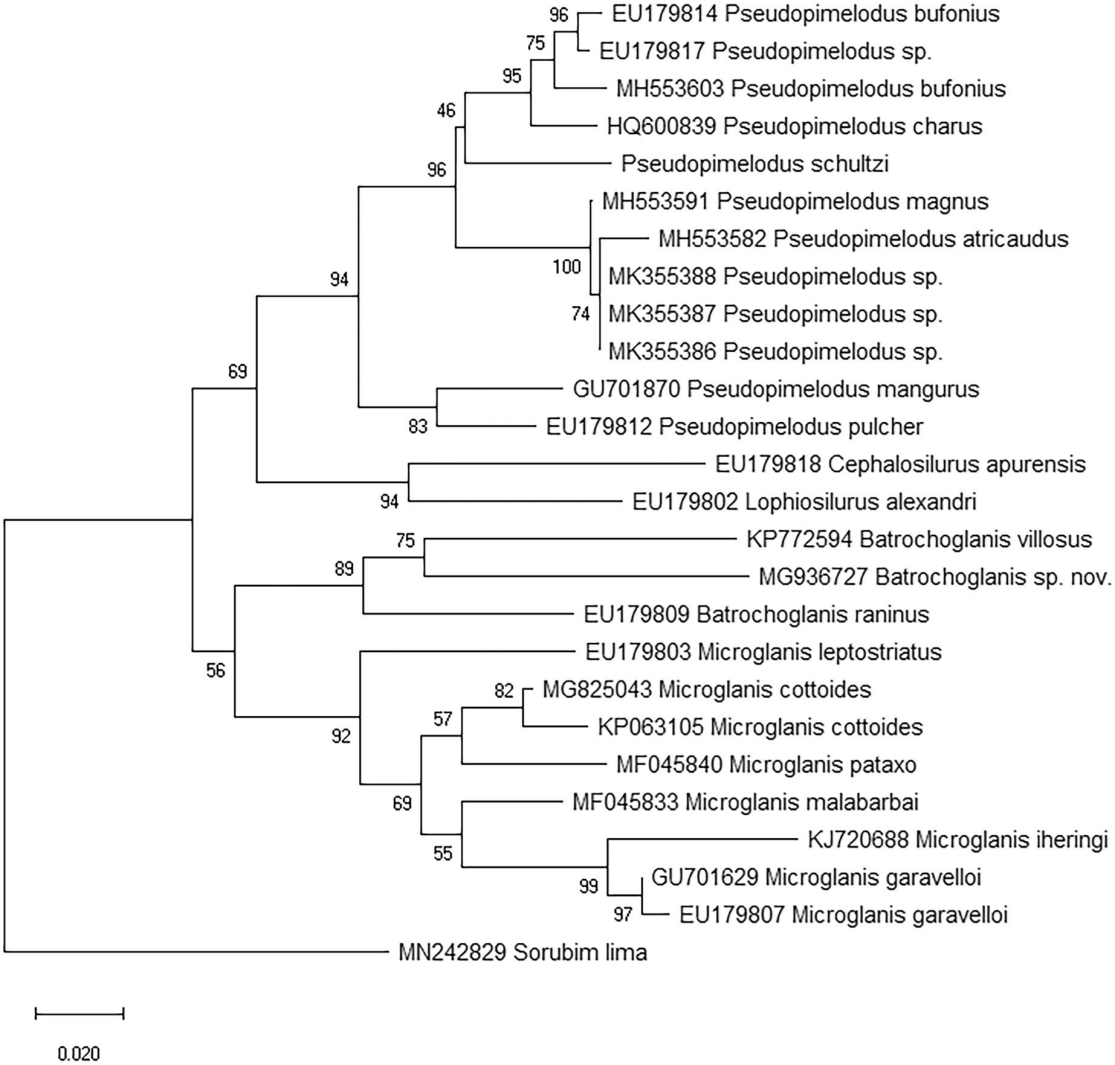
Neighbour-Joining phylogenetic tree of the *P. schultzi* and other species based on the cytochrome c oxidase subunit I (COI) gene. Numbers on nodes indicate bootstrap support value, based on 1000 replicates.

## Data Availability

The genome sequence data that support the findings of this study are openly available in NCBI at https://www.ncbi.nlm.nih.gov under the accession number MW959699, The associated BioProject, SRA, and Bio-Sample numbers are PRJNA734428, SRR14705851, and SAMN19491011 respectively, or available from the corresponding author.
